# A Comparative Study of Relationship between Micronutrients and Gestational Diabetes

**DOI:** 10.5402/2012/470419

**Published:** 2012-09-04

**Authors:** Farideh Akhlaghi, Seyyed Majid Bagheri, Omid Rajabi

**Affiliations:** ^1^Obsterics and Gynecology Department, Women Health Research Center, Faculty of Medicine, Mashhad University of Medical Sciences, Mashhad 9137913316, Iran; ^2^Omalbanin Hospital, Azadi Street, Mashhad 9144663595, Iran; ^3^Department of Physiology, Faculty of Medicine, Yazd University of Medical Sciences, Yazd 8944157963, Iran; ^4^Pharmaceutical Chemistry Department, Faculty of Pharmacy, Mashhad University of Medical Sciences, Mashhad 917751365, Iran

## Abstract

In this paper, we studied the relation between the micronutrient and gestational diabetes. Therefore, we measured micronutrient concentration including Ni, Al, Cr, Mg, Fe, Zn, Cu, and Se in serum of women with gestational diabetes between 24 and 28 weeks of gestational age (study group) who had inclusion criteria and comparison with micronutrient levels in normal pregnant women with same gestational age (control group). Results showed that there was no significant difference between the serum micronutrient level (Ni, Al, Cr, Mg, Zn, Cu, Se) in study and control groups except serum level of iron which in serum of gestational diabetic women was lower than normal pregnant women and difference was significant.

## 1. Introduction

Gestational diabetes mellitus (GDM) is characterized by glucose intolerance during pregnancy. Initiation of GDM usually is in middle and late gestational period and continues to term [1, 2]. The etiology of GDM is not yet clear. Approximately 1–14% of all pregnancies are complicated by GDM [3, 4]. For prevention of these adverse effects, screening test between 24 and 28 weeks of gestation was performed and with this manner though 40% of women with GDM could be detected early during pregnancy [5–7]. There are several predisposing factors for gestational diabetes such as age, obesity, and weight gain (body mass index > 25 GDM) in previous pregnancy or history of familial diabetes [6, 8]. Previous researches also had shown that some micronutrients similar to chromium have important roles in developing to diabetes [7, 9–11]. Selenium [Se] is an essential micronutrient and plays important role in human biology. Selenium exerts its biological effect through over 30 selenoproteins detected in mammalian systems [11–16]. In this research we decided to study relation between level of micronutrients in serum and others nutrition factors with gestational diabetic women and comparison with normal pregnant women. 

## 2. Materials and Methods

### 2.1. Subjects

Women who were primigravid, between 24 and 28 weeks of pregnancy age and cared for in prenatal care clinic of Emam Reza hospital participated in this study. For those who satisfied and signed the satisfaction forms for participation in this study, glucose challenge test was done. In women who had abnormal glucose challenge test (plasma glucose level ≥130 mg/dL but lower than 200 after 1 hour of eating 50 gram oral glucose) oral glucose tolerance tests (OGTT) were done. For performing OGTT, we asked that all of participants must have an unrestricted diet (containing at least 150 g carbohydrate per day) for 3 days before test. On the day of test, the following steps were done.

First a blood sample was collected after 8–10 hours fasting. Then they drank a solution containing a 100 gr of glucose, and drawing blood to measure glucose levels 1, 2, and 3 hours after that was done. The following values as statement of American Diabetes Association considered as abnormal value areas follows.

- Fasting blood glucose level ≥95 mg/dL (5.33 mmol/L).
- 1 hour blood glucose level ≥180 mg/dL (10 mmol/L).
- 2 hour blood glucose level ≥155 mg/dL (8.6 mmol/L).
- 3 hour blood glucose level ≥140 mg/dL (7.8 mmol/L).

In women who had abnormal OGTT (2 abnormal values of 4 plasma glucose levels), OGTT was repeated 1 week after the initial test for rule out false positive tests. Those who had abnormal second OGTT too were selected as gestational diabetic group (study group). Pregnant women who underwent OGTT during the same period and had normal oral glucose tolerance test were included as normal group (control group). In each group 30 women who were matched with each other were selected as study and control groups. This study was reviewed and approved by the Institutional Review Board of Vice Researches in Mashhad University of Medical Sciences. In all participants, history of familial diabetes mellitus (paternal/maternal), education level (according to their statements), and previous macrosomic neonates delivery were asked and filled in the questionnaire forms. In all participants body mass index (BMI) (weight in kilograms divided by square of height in meter) and waist (waist circumference in centimeter by placing a tape measure around the bodies at the top of their hipbone) were calculated.

### 2.2. Biochemical Analysis

Plasma samples were collected and stored at −20°C in the Pharmaceutical Chemistry Department of Mashhad University of Medical Sciences. After all samples were collected, measurements of nickel, aluminium, chromium, magnesium, iron, zinc, copper, and selenium were done by atomic absorption. Then standard diagram was drawn for each of cations and validation was done and micronutrients were measured in each sample. Sensitivity of methods for determination of micronutrients was 1 ppm. All data were analyzed by graph pad prism software version 5 and comparison between study and control groups.

### 2.3. Statistical Analysis

All data are presented as mean ± standard deviation. Statistical analysis was performed by using the graph pad prism 5. We compared characteristics between two groups by using *t*-test. 

## 3. Results

The clinical characteristics of GDM group and control group are summarized in Table 1. In Study group advanced age, greater BMI, and waist in comparison with control group had significant differences (*P* < 0.05). In study group there were positive relations with gestational diabetes and previous childbirth macrosomic neonates (≥4000 gr) and history of familial diabetes. Control group was educated higher than study group. There was no significant difference between blood pressures of study group and control group.  Table 2 has shown the serum concentration of micronutrients including nickel, aluminum, chromium, magnesium, iron, zinc, copper, and selenium in study group and control group. As shown, there is no significant difference in concentration of micronutrients between the study group and control group except iron concentration in study group was significantly lower than control group.

## 4. Discussion

The specific aim of this study was to pursue possible relationships between serum concentration levels of several micronutrients and typical indicators used in the followup of gestational diabetic women, thus contributing to better understanding of the global role of micronutrients in GDM complications. GDM is a complex metabolic syndrome and many factors are contributed in this disorder. The causes of GDM are multifactorial and may include genetic and environmental factors that influence insulin sensitivity. Our data showed that age, BMI, and familial history of diabetes were higher in study group in comparison with control group. GDM is directly proportional to maternal body mass index. Previous study showed that incidence of GDM in pregnancy in women who are obese is higher than that of the general obstetric population [11, 13]. On the other hand, weight gain during pregnancy and increasing maternal age worsen the risk of developing GDM [14, 15].

Risk factors include high BMI, previous macrosomic neonates, previous GDM, and familial history of diabetes have high prevalence of diabetes and should alert the clinician to screen those pregnant women for GDM. Our current study supports this finding for gestational diabetic women which in whom age, BMI, familial history of diabetes, previous history of macrosomia neonates are significant differences in comparison with control group. Previous GDM is one of the strongest predictors for GDM [16]. One factor which influences the prevalence of diabetes mellitus is socioeconomic and education level status. In our study, lower education status was associated with the development of GDM. It is possible that this is related to a traditional diet which is inexpensive but has a high fat and carbohydrate contents. In addition previous studies have shown that some micronutrients have important role in diabetes mellitus and consumption of some trace elements can improve effects of diabetes mellitus [17]. Chromium is thought to play a key role in normal carbohydrate metabolism by potentiating the action of insulin, leading to increase insulin sensitivity in type II diabetes and obesity [18]. Some studies have focused on a single element in order to understand its specific biological role, yet multielemental determinations have also been carried out [19]. The results reported in the above mentioned studies indicate higher serum concentration levels of copper, lead, arsenic, cadmium, nickel, aluminum, and lower levels of selenium, chromium, manganese, and zinc in diabetic patients than healthy individuals. Several micronutrients have beneficial effects in healthy individuals and also in diabetic patients [20]. In particular, copper, zinc, selenium, iron, or manganese are essential components of metal enzymes such as Se containing glutathione peroxidase, Cu/Fe cytochrome C oxidase, or different types of superoxide dismutases; all of them are important in intra- and extracellular antioxidant defense. On the other hand, some metallic species are considered important in glucose metabolism. For example, trivalent chromium was proposed as a structural part of glucose tolerance factor and even though such compound has not been confirmed, it is believed that Cr participates in stimulation of insulin signaling [21]. Other elements to be mentioned is zinc, which is necessary for the synthesis of insulin hexamer [22]. Vanadium salts have been used to lower glucose levels in diabetes patients even before the discovery of insulin; however the molecular mechanisms underlying the observed effects are not clear [23]. Some studies were done on the relation between Iron status and diabetes. They suggested that iron may play a role in the pathogenesis of type 2 diabetes because iron is a strong prooxidant and high body iron levels are associated with increased level of oxidative stress that may elevate the risk of type 2 diabetes [24]. So some researchers studied the relation between iron supplementation in pregnancy and diabetes. One of those studies concluded iron supplementation is associated with glucose impairment and hypertension in midpregnancy [25]. In contrast another study concluded that there was no significant difference in the incidence of GDM in iron supplement and placebo groups at 28 weeks and iron supplement from early pregnancy does not increase the risk of GDM [26]. In one study which was performed to compare iron status in GDM and control group and their findings indicated an association between increased iron status and GDM [27]. But results of our study showed significant association between low iron level and GDM. Therefore such studies are required to prove the causal relationship between iron level and gestational diabetes. 

## 5. Conclusion

Advanced age, high BMI and waist, and familial history of diabetes had significant differences between study and control groups. Relation between micronutrients including Ni, Al, Cr, Mg, Zn, Cu, Se, and gestational diabetes had not significant differences in two groups, except iron level which in serum of gestational diabetic group was lower than normal group and difference was significant. 

## Figures and Tables

**Table 1:** Clinical characteristics of participants in study of relationship between micronutrients and gestational diabetes.

Subjects	Normal group	GDM group	*P*
Age (year)	25	30	<0.05
BMI (kg/m^2^)	28	31	<0.05
Family history of diabetes	30%	50%	<0.05
Macrosomia (≥4000 gr)	0%	3.3%	ns
Diastolic pressure (mmHg)	6.6	6.1	ns
Systolic pressure (mmHg)	10.5	10.7	ns
Waist (cm)	101.3	112.6	<0.05

**Table 2:** Micronutrients level in serum of participants in study of relationship between micronutrients and gestational diabetes.

Micronutrients	Control group	GDM group	*P*
Se (ng/mL)	134.33	137.43	ns
Cu (mcg/mL)	0.91	0.93	ns
Zn (mcg/mL)	0.82	0.88	ns
Fe (mcg/mL)	85.51	73.25	<0.05
Mg (mg/dL)	1.65	1.67	ns
Cr (mcg/L)	0.33	0.31	ns
Al (mcg/L)	0.15	0.12	ns
Sn (ng/mL)	30.7	30.6	ns
Ni (mcg/mL)	0.91	0.99	ns
